# Low Cost AIP Design in 5G Flexible Antenna Phase Array System Application

**DOI:** 10.3390/mi11090851

**Published:** 2020-09-13

**Authors:** Wei-Shin Tung, Wei-Yuan Chiang, Chih-Kai Liu, Chiung-An Chen, Pei-Zong Rao, Patricia Angela R. Abu, Wan-Ming Chen, Faisal Asadi, Shih-Lun Chen

**Affiliations:** 1 Shenzhen Jaguar Wave Technology Co. Ltd., Shenzhen 518103, China; Weishin.tung@jaguarwave.com (W.-S.T.); chihkai.liu@jaguarwave.com (C.-K.L.); peizong.rao@jaguarwave.com (P.-Z.R.); Maxwell.chen@jaguarwave.com (W.-M.C.); 2 Department of Electrical Engineering, Ming Chi University of Technology, New Taipei City 24301, Taiwan; asadifaisal@gmail.com; 3 Department of Information Systems and Computer Science, Ateneo de Manila University, Quezon City 1108, Philippines; pabu@ateneo.edu; 4 Department of Electronic Engineering, Chung Yuan Christian University, Taoyuan City 32023, Taiwan

**Keywords:** phase array antenna, antenna in package, 28 GHz antenna

## Abstract

In this paper, a low cost 28 GHz Antenna-in-Package (AIP) for a 5G communication system is designed and investigated. The antenna is implemented on a low-cost FR4 substrate with a phase shift control integrated circuit, AnokiWave phasor integrated circuit (IC). The unit cell where the array antenna and IC are integrated in the same plate constructs a flexible phase array system. Using the AIP unit cell, the desired antenna array can be created, such as 2 × 8, 8 × 8 or 2 × 64 arrays. The study design proposed in this study is a 2 × 2 unit cell structure with dimensions of 18 mm × 14 mm × 0.71 mm. The return loss at a 10 dB bandwidth is 26.5–29.5 GHz while the peak gain of the unit cell achieved 14.4 dBi at 28 GHz.

## 1. Introduction

Antenna technology is the latest breakthrough in design to accelerate cellular networks that aims to optimize the communication system in terms of smoothness and cost of the communications itself. Along with the development of the cellular network, currently the newest generation technology of the networks has arrived at the fifth generation (5G) network. The success of the 5G network paved its way to research for the newest technology product and to provide the best communication platform [1,2]. The 5G internet has become the center of research on how to improve its capabilities and novelty of the technology itself. The design of the Antenna in Package (AIP) is one field of research of 5G technology that can be improved to maximize the capabilities and functionalities of the technology [3].

The proposed research aims to optimize the bandwidth capabilities of the 5G technology by designing the AIP with models that exhibit low-cost array antenna design. The recent research on this field performed optimization of the 5G technology through space-frequency index modulation, spectral, energy, and economic fields [4,5]. On the other hand, many research also explored optimizing the scalability of the bandwidth of the 5G communication network. The first research was from K. Kibaroglu et al. [6] that has successfully designed a simple model 32-element (4 × 8) working at 28 GHz on the phased-array transceiver for 5G communication technology based on a 2 × 2 beamformer core chips. The research has achieved an effective isotropic radiated power (EIRP) of 43 dBm at P1dB, and the final state-of-art data rate was achieved in 1.0–1.6 Gb/s in a single beam using 16-QAM.

Similar research on improving the 5G technology network in a different area is conducted by J. Park et al. They improved the concept of the 5G technology through the use of the invisible Antenna-on-Display (AoD) that has been successful in the millimeter-wave for the cellular network. The invisible concept was designed and fabricated in a 1 × 8 optically invisible array that exhibits a 66.6 dBi boresight gain that operate at 28 GHz, which is still capable of maintaining 88% of the optical transparency. On the other side, A. M. Pawan Kumar et al. designed a quad-port wide-band multiple-input-multiple-output (MIMO) that integrated the wide Axial-ratio concept. This was successfully designed in a FR-4 dielectric substrate with size 45 × 45 × 1.6 mm^3^. Moreover, the design of the proposed MIMO concept showed a 3-dB ARBW of 52% (3.8–6.5 GHz) and an impedance bandwidth (S11 ≤ −10 dB) of 144% (2.2–13.5 GHz) [7,8].

G. F. Hamberger et al. proposed an antenna array with a planar dual-polarized microstrip 1-D-Beamforming for the 24 GHz band. Simulation results showed that it could operate in a frequency of over 500 MHz. Similar work proposed a power-efficient multiband planar USB dongle antenna for a wireless sensors network. A USB dongle antenna was designed to work with three frequencies bands namely, 2.30–2.69 GHz, 3.40–3.70 GHz, and 5.15–5.85 GHz. At the end of the research, the efficiency of power consumption in the looping process has significantly improved [9,10,11].

The spread of virus and influenza in recent years required the monitoring of physiological signals without contact to the subjects, which is of the utmost priority. The wireless body sensor networks (WBSNs) [12,13] overcome the difficulties of high risk infecting. Furthermore, C.A. Chen et al. provided a low power [14] and efficient compression algorithm [15] to increase the effectiveness of communication data without any loss. Body signals with noise are easy to confuse the diagnosis and misjudge the symptom is another challenge. A filter with a reconfigurable clock [16] was designed for WBSNs with better noise filtering therefore acquiring smooth signals. With the advancement of both medical imaging and compressors, S.H. Chen et al. [17,18] used fuzzy decision and resolution to improve the rate of image compression. Moreover, a central control unit and cost-efficient WBSNs systems were required in a micro control unit implementation [19]. Moreover, the modularized device brings a lot of convenience on combining the system and can simplify the design of many other functional devices, like the wireless transmission of medical data by a wearable device [20]. These previous works contributed to the efficiency of the designs and real-time data implementations to wireless communication devices.

This paper expands and continues previous studies and proposes an improvement in the design of the AIP that is a low-cost 28 GHz AIP for the 5G communication system that is based on 2 × 2 beamformer core chips. The next sections present the phased-array architecture of the unit cells, the analysis system for the elements array, and circuit blocks. Section 4 presents and discusses the results and performance of the proposed design. In this study, the frequency band of the antenna focuses on the n257 band (26.5–29.5 GHz) [21].

## 2. Patch Antenna Design

In this study, the micro strip patch antennas constructed the array system. The patch antenna is a kind of a resonant antenna that is like a resonant cavity. One important parameter of a resonant cavity is its quality factor ($Q_{0}$), which is defined as shown in Equation (1) [22].

(1) $$Q_{0}\equiv \omega \frac{2W_{e}}{P_{l}}$$

where ω is the frequency, $W_{e}$ is the stored energy in the resonant cavity, and $P_{l}$ is the power loss of the resonant cavity. There are three kinds of losses in the resonant antenna namely radiation loss ($P_{rad}$), dielectric loss ($P_{d}$), and conducted loss ($P_{c}$). The formula is shown in Equation (2).

(2) $$\frac{1}{Q_{0}}=\frac{P_{rad}}{2\omega W_{e}}+\frac{P_{d}}{2\omega W_{e}}+\frac{P_{c}}{2\omega W_{e}}=\frac{1}{Q_{rad}}+\frac{1}{Q_{d}}+\frac{1}{Q_{c}}$$

The antenna efficiency can be enhanced when the dielectric loss is reduced. This is due to the antenna efficiency (ξ), as shown in Equation (3) that is proportional to $Q_{0}$ when the conducted loss is fixed in the critical coupled condition.

(3) $$\xi =\frac{P_{rad}}{P_{l}}\propto \frac{Q_{0}}{Q_{rad}}$$

**Figure 1:**
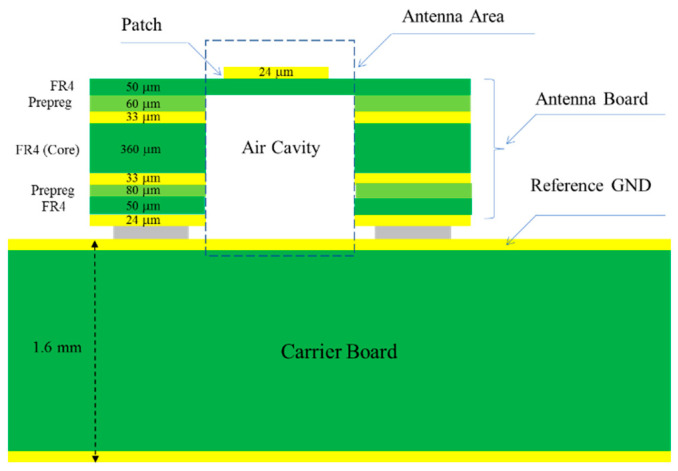
The structure of the patch antenna with air cavity.

**Figure 2:**
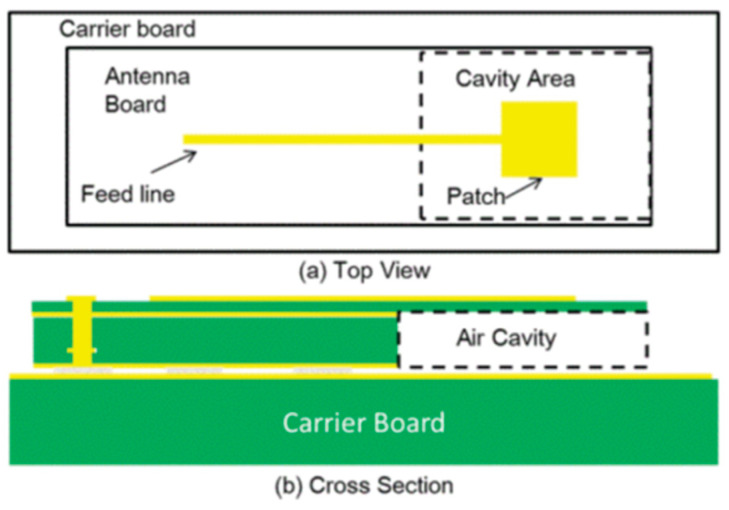
(**a**) Top view and (**b**) cross section view of the patch antenna.

This study used an air-filled cavity structure to design the patch antenna on a standard FR4 substrate [23]. This design constructed a metal patch that was located on the FR4 substrate with the open air cavity. The reference ground used the copper layer on the carrier board as illustrated in Figure 1. This design can reduce the dielectric loss and enhance the patch antenna performance with better radiation efficiency. The dielectric constant of air was 1.0006 and the loss tangent of air was 0, which can enhance the patch antenna performance with better radiation efficiency. The top and cross section views are shown in Figure 2.

**Figure 3:**
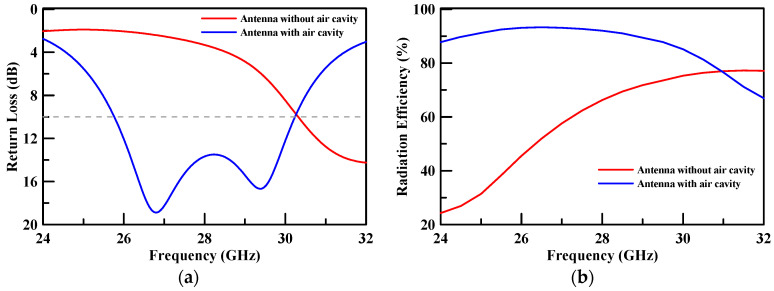
(**a**) The return loss and (**b**) the radiation efficiency of the patch antenna.

The return loss and radiation efficiency of the patch antenna are presented in Figure 3. Figure 3a illustrates that the return loss of the patch antenna with air cavity is better than 10 dB at 26.5–29.5 GHz. Figure 3b presents the radiation efficiency of the two types of antenna where the radiation efficiency of the patch antenna with air cavity was 92% while the radiation efficiency of patch antenna without air cavity was 66.25% at 28 GHz. The radiation efficiency was enhanced by 25.75% at 28 GHz. The maximum radiation efficiency of the patch antenna with air cavity was 93.28% while the maximum radiation efficiency of patch antenna without air cavity was 77.25%. The radiation efficiency was enhanced by 16.03%.

## 3. Array Antenna Design

**Figure 4:**
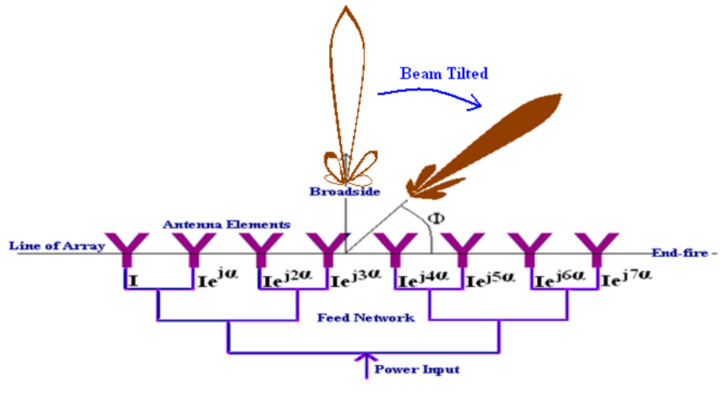
Beam steering/scanning antenna array [24].

The operation frequency band of the 5G system achieves the Ka-band. A small wavelength, small beam width, and high atmospheric attenuation are the shortcomings of this frequency band while its great advantages are its larger bandwidth and higher data rate. The multiple antenna techniques (MTA) is the solution that can solve wave shadowing of millimeter wave propagation [24]. The array antenna is an important development. The array antenna is composed of antennas that are arranged periodically as illustrated in Figure 4. The beam main lobe can be tilted by changing the phase of the antennas, which is called the beam steering technique.

**Figure 5:**
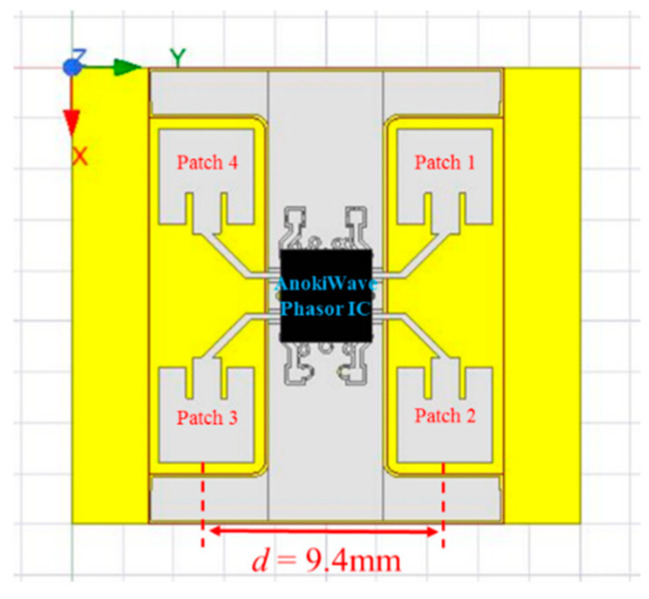
The structure of proposed array antenna with phasor IC.

In this study, the four patch antennas constituted a 2 × 2 array antenna as shown in Figure 5. An AnokiWave phasor IC was set at the same side with the patch antennas. Such an arrangement makes the array antenna become a complete system. This modular system is more flexible and expandable, which is widely known as the Antenna-in-Package (AIP).

**Figure 6:**
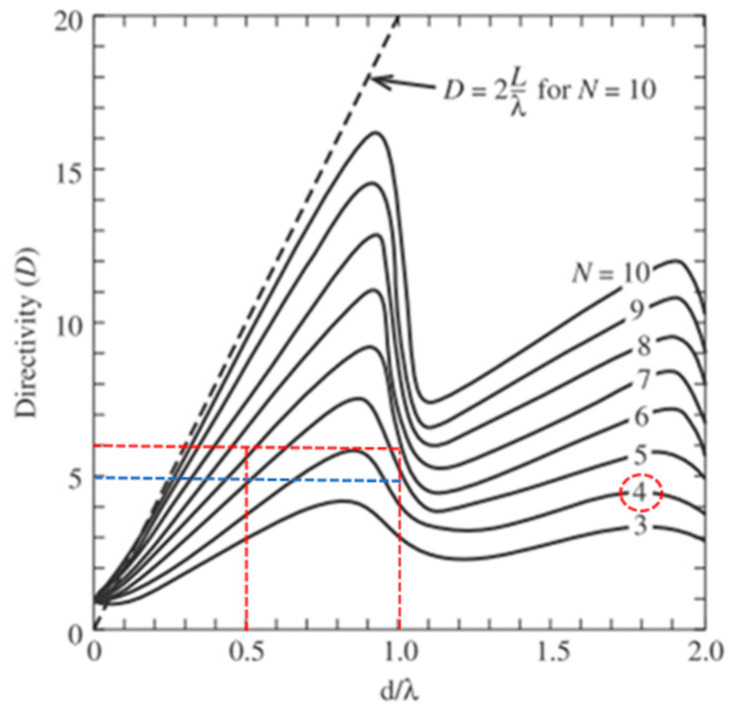
Directivity as a function of antenna spacing for a broadside array of isotropic elements [25].

The antenna spacing *d* is an important parameter in the design of the array antenna. In Figure 6, the ideal maximum array directivity (*D*) of a 2 × 2 array antenna is 6 dBi [25]. Basically, the single antenna gain (*G*) as shown in Equation (4) is proportional to the directivity of a single antenna. In fact, the antenna efficiency of each element does not need to be considered when taking into account the array gain. The array gain is equal to the array directivity. In this study, the estimated array gain is 5–6 dBi. Otherwise, the maximum scan angle must satisfy the condition in Equation (5). The $\theta_{max}$ is the maximum angle to which the array can be steered. The steering can be reckoned by Equation (5). The maximum angle is listed in Table 1 with an operating frequency of 28 GHz.

(4) $$G_{single\;antnna}=\xi \cdot D_{single\;antenna}$$

(5) $$\frac{d}{\lambda }\leq \frac{1}{1+\left|\sin\theta_{max}\right|}$$

The ideally maximum steering of the array antenna was ±90°. With that the antenna spacing was half the wavelength. In this study, the minimum spacing was 9.4 mm since the phasor IC was set at the center of the proposed array antenna. The maximum steering of the proposed array antenna approached ±10°.

**Figure 7:**
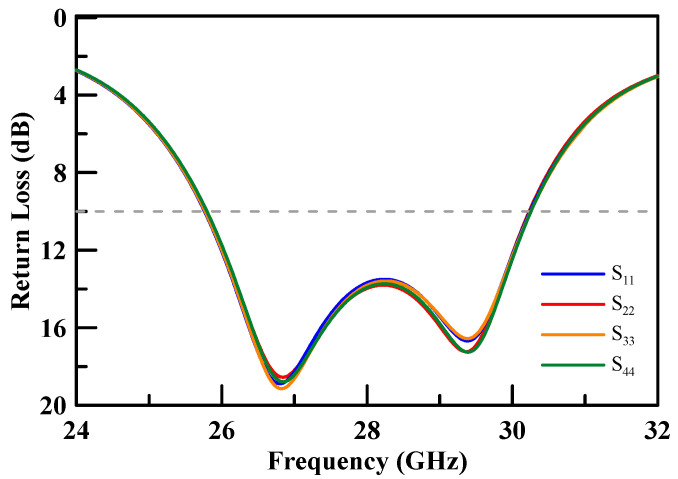
Four (4) ports return loss of the simulation.

**Figure 8:**
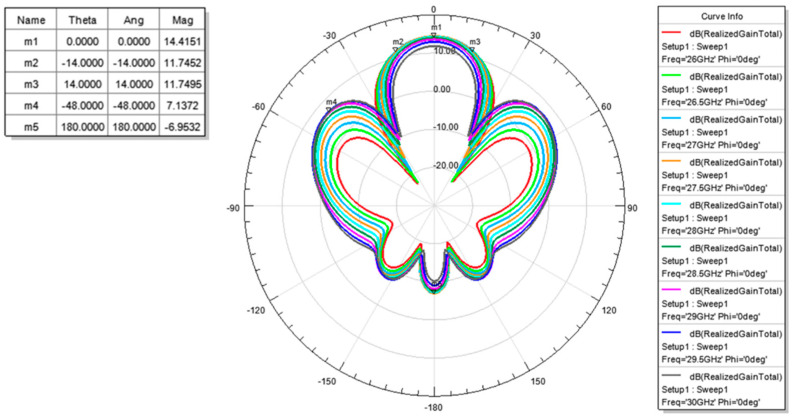
Radiation pattern of the simulation (X Cut).

**Figure 9:**
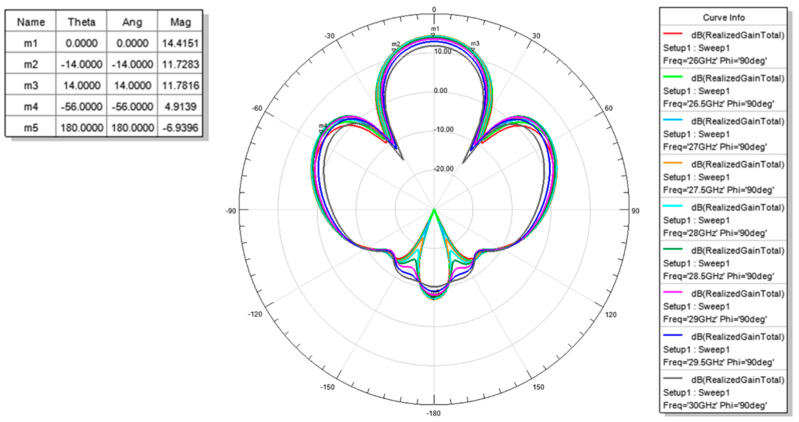
Radiation pattern of the simulation (Y Cut).

**Figure 10:**
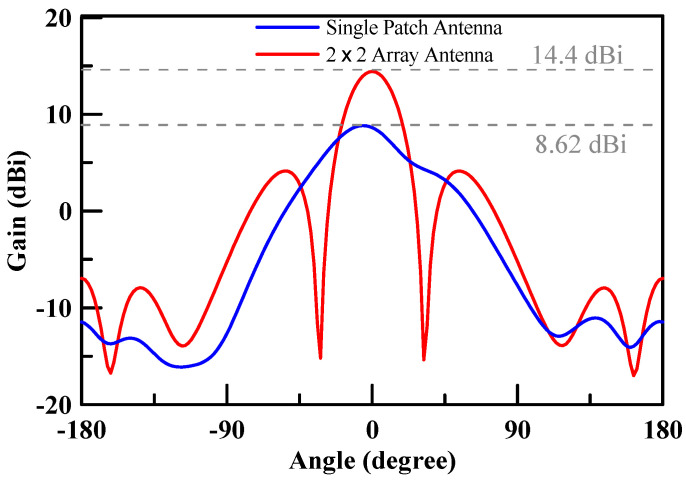
Antenna peak gain.

The measured return loss of the simulated 2 × 2 array antenna of each port was better than 10 dB at an operating frequency of 26.5–29.5 GHz as shown Figure 7. The simulation results of each port were highly consistent, which is due to the structure of the array antenna that is in symmetry. The antenna peak gain was 14.4 dBi as shown through m1 in Figure 8 and Figure 9. The 3 dB beam width that is shown through m2 and m3 on Figure 8 and Figure 9, respectively, was 26°. The comparison of the simulation results of the single antenna and the array antenna is shown in Figure 10. The array gain was 5.78 dB, which was consistent with the estimative value.

**Figure 11:**
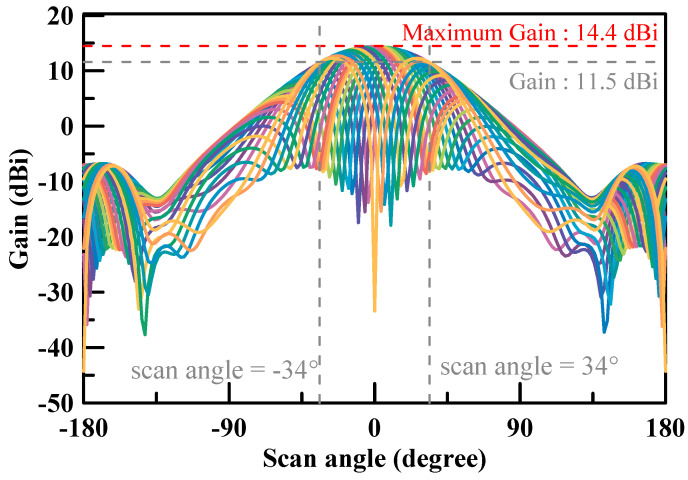
Simulation results of the beam steering pattern at 28 GHz (X-cut).

**Figure 12:**
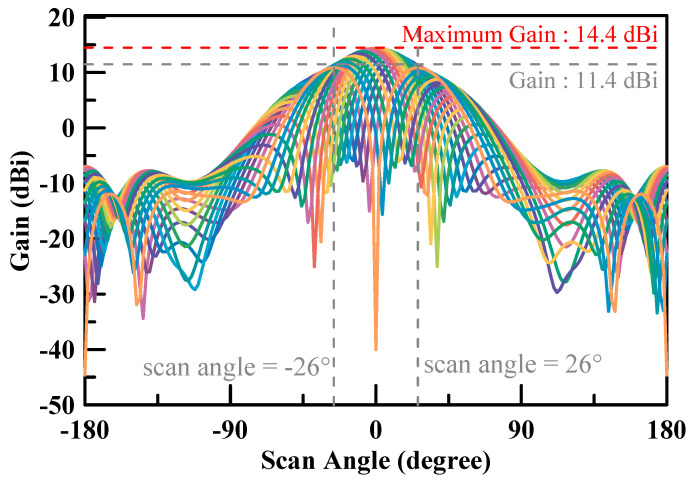
Simulation results of the beam steering pattern at 28 GHz (Y-cut).

The filed pattern of beam steering can be simulated by changing the phase of the four patch antennas. The simulation results of the beam steering tilted angle at 28 GHz are shown in Figure 11 and Figure 12. The maximum gain was 14.4 dBi for both X cut and Y cut. The beam steering tilted angle was ±34° in the X cut while the beam steering tilted angle was ±26° in the Y cut.

## 4. Antenna Manufacturing and Experimental Measurement

Progressive and lower loss materials were used to design a millimeter-wave antenna, such as Rogers (RO 4003C or RO 4350B), low temperature co-fired ceramics (LTCC), PTFE, and liquid crystal polymer (LCP). The manufacturing process of these novel kinds of materials is complex and their manufacturing costs are very expensive. The FR4 substrate has a lower cost compared to the other kinds of materials. The material cost of a Rogers material is three to five times more expensive than that of an FR4 material. Furthermore, the choice of the manufacturer, manufacturing quantity, design metal layers, and ordering options also affect the overall cost of the whole process. On the other hand, a low loss material process is 100 times more expensive than the manufacturing cost of a traditional FR4 PCB. However, the loss tangent of the low cost FR4 material is 0.01–0.04 at a frequency band of 26.5–29.5GHz, which restricts the performance of the antenna. The gain of the antenna that is designed on an FR4 substrate is approximately 4.5 dBi. The performance of the antenna that is designed on an FR4 substrate can be enhanced by using the air-filled cavity structure.

**Figure 13:**
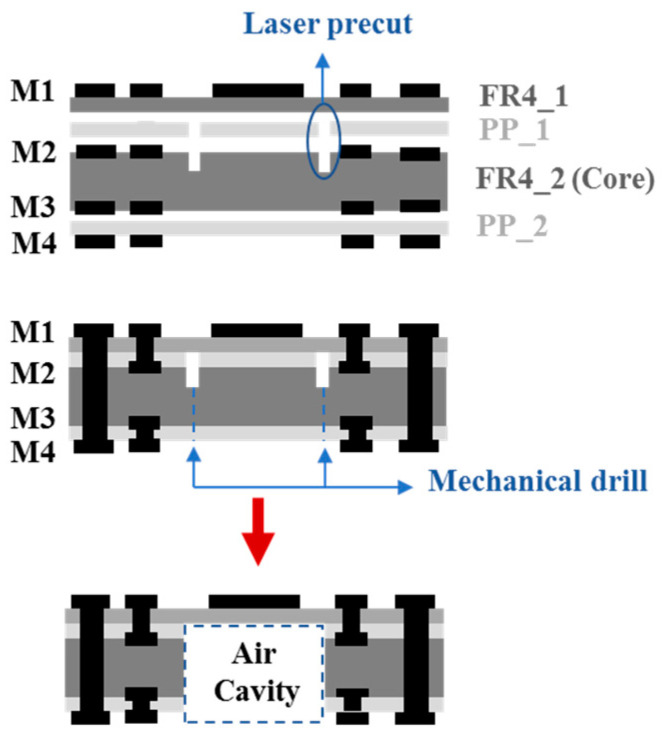
Manufacturing process of the antenna with an air-filled cavity.

The antenna module proposed in this study was designed with a stack of three substrates and four metal layers (M1, M2, M3, and M4) as illustrated in Figure 13. The production process started by completing the circuit etching of the middle layer (M2 and M3) followed by the addition of two layers of PP (PP_1 and PP_2) on top and below the middle layer. During this step, the upper and lower materials of M2 (PP_1 and FR4_2) were laser precut as shown in the figure. The purpose of the laser precut is to leave a cutting path that will be used as a guide for the removal of the center substrate area later in the process. The next step was the lamination of M1 and M4, and the circuit etching for both metal layers. This was followed by creating laser holes from M1 to M2 and M3 to M4, and finally from M1 to M4. The final step involved mechanical drilling at the M4 surface towards the laser precut. Once the holes from the M4 surface to the laser precut were properly drilled and aligned, the center substrate could be removed therefore exposing the area of the entire air cavity. The key point of the process technology is on the air-filled cavity structure. The tolerance of each air-filled cavity must be made as small as possible. If the tolerance turned out to be significantly large, it will lead to a significant difference in the gain of each patch antenna. In turn, the performance of the array will be affected. In addition, the reserved M1 layer and its supporting material FR4_1 must be designed to be thin in order to have a lossless air-filled cavity. Moreover, if the air-filled cavity is too large in terms of area, it will have an impact on the antenna gain due to the changed distance of the patch relative to the ground.

**Figure 14:**
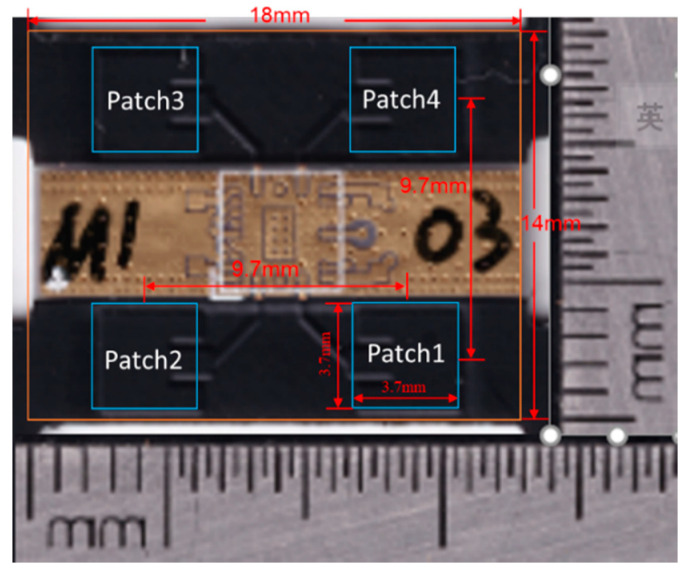
Photograph of the array antenna (2 × 2).

**Figure 15:**
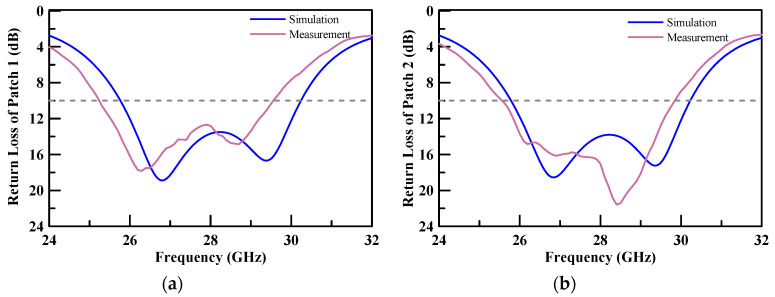

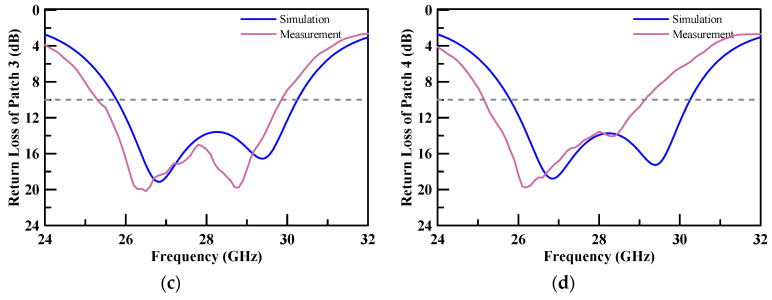
Comparison of the simulation and empirical results of the return loss of each patch, (**a**) return loss of Patch1; (**b**) return loss of Patch2; (**c**) return loss of Patch3; (**d**) return loss of Patch4.

The proposed array antenna was manufactured on an FR4 substrate. Figure 14 shows a photograph of the array antenna assembly. The measured results of the return loss for each port were better than 10 dB at an operating frequency band of 26.5–29.5 GHz. The comparison of the simulation and empirical results are presented in Figure 15. The empirical results are shown to satisfy the requirement of a 5G system millimeter wave band.

**Figure 16:**
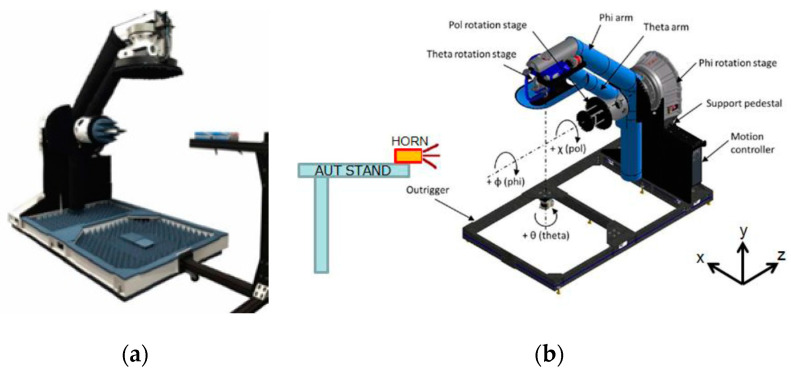
NSI-700S-360 antenna chamber, (**a**) instrument diagram; (**b**) equipment setup [26].

**Figure 17:**
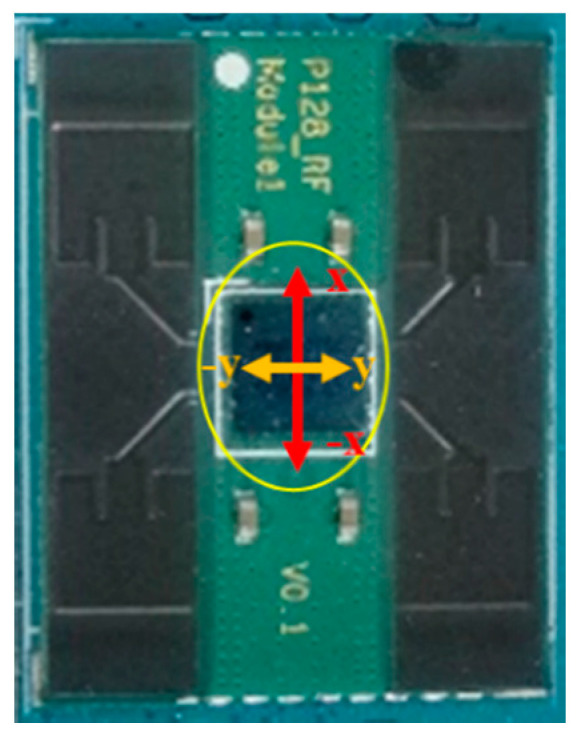
Measurement coordinates.

**Figure 18:**
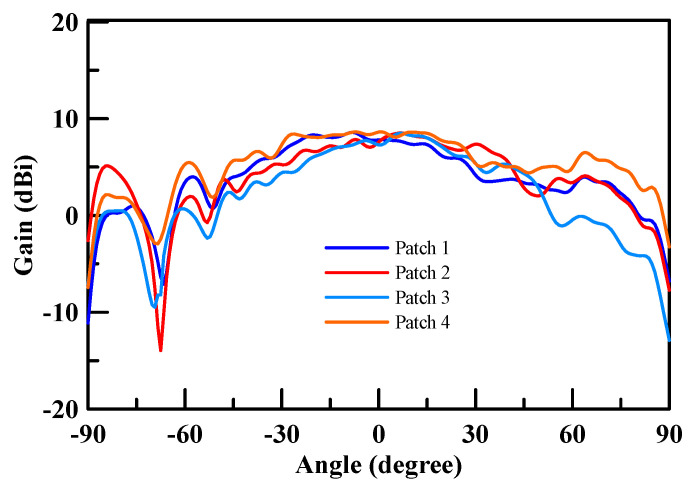
Single patch antenna gain (X-cut).

**Figure 19:**
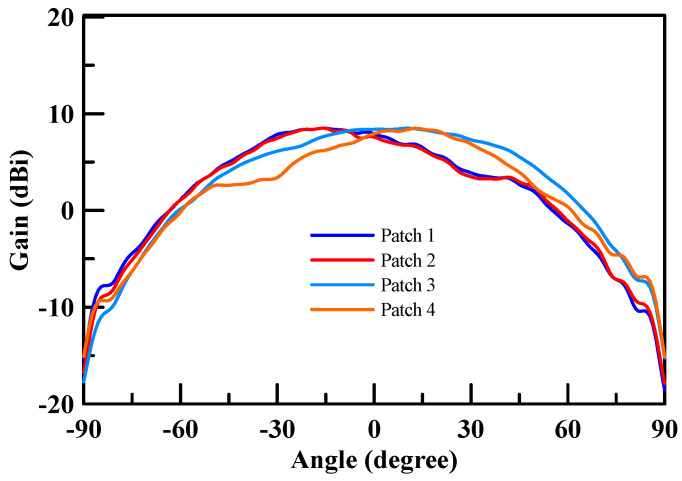
Single patch antenna gain (Y-cut).

Figure 16 shows an NSI-700S-360 antenna chamber [26]. Its measurement coordinates are shown in Figure 17. The gain measurement results of each patch antenna are shown in Figure 18 (X-cut) and Figure 19 (Y-cut). The maximum gains were 8.58 dBi for Patch 1, 8.47 dBi for Patch 2, 8.49 dBi for Patch 3, and 8.64 dBi for Patch 4 in the X-cut. The maximum gain was 8.5 dBi for each Patch antenna in the Y-cut.

**Figure 20:**
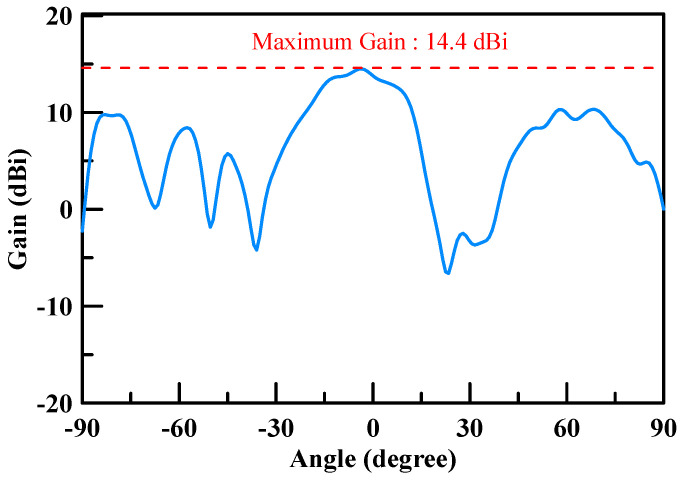
Array antenna (2 × 2) gain measurement results (phase 0/180/180/0, X-cut).

**Figure 21:**
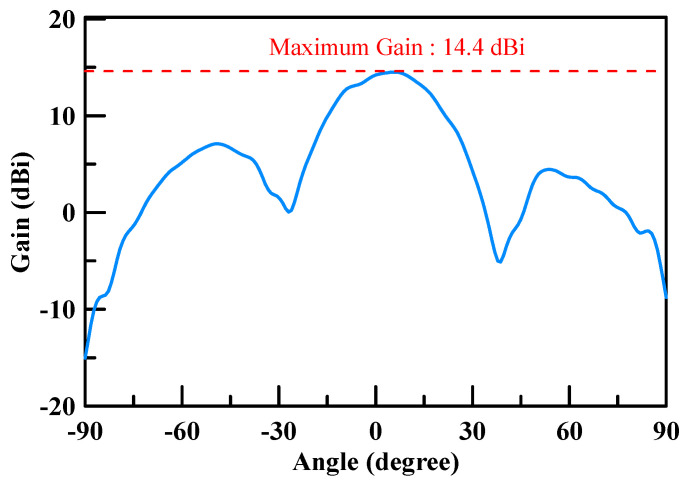
Array antenna (2 × 2) gain measurement results (phase 0/180/180/0, Y-cut).

**Figure 22:**
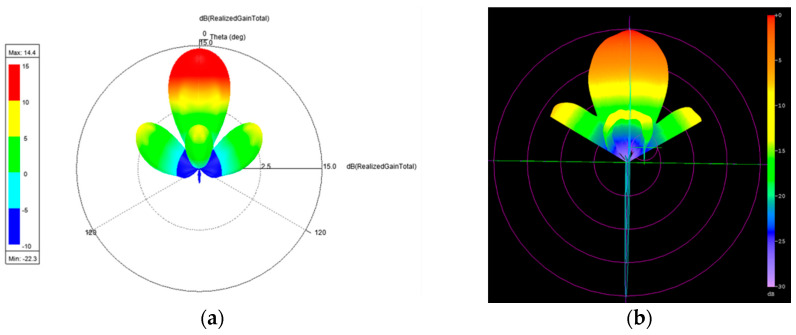
3D radiation pattern of the array antenna at 28 GHz: (**a**) simulation result and (**b**) measurement result (normalized).

The gain measurement results of the array antenna are shown in Figure 20 and Figure 21. The maximum gain was 14.4 dBi for the two cuts. The operated conditions of the phase for each patch antenna were Patch 1: 0 degree, Patch 2: 180 degree, Patch 3: 180 degree, and Patch 4: 0 degree. These results conform to the principle presented in Section 2. The 3D normalized radiation pattern is shown in Figure 22b, which shows similar 3D radiation patterns to the simulation results shown in Figure 22a.

## 5. Conclusions

The design and simulation of a 2 × 2 low cost phase array antenna module for 5G applications operating at 28 GHz with 14.4 dBi antenna gain was proposed in this paper. The air-filled cavity used for patch antenna structure was with a FR4 PCB material for cost reduction instead of using a Roger or M6 material PCB. Moreover, it improved the antenna radiation efficiency by reducing the loss of the material. Furthermore, the designed array unit could be used and combined for a higher order array along two dimensions with a suitable surface mount technology (SMT) gap. It helps to easily and reliably implement a high order array. Therefore, the proposed array antenna is a promising candidate for the mm-wave 5G small cell applications. Table 2 summarizes the performance of this work and compares it with state-of-the-art mm-wave phased-array antennas [27,28,29,30,31,32,33,34,35,36,37,38]. The proposed patch shows around an 8.5 dBi antenna gain, which is better than [31,35,37,38], at a similar frequency. It describes that the air-filled cavity as a patch gap between the ground increased the antenna efficiency effectively instead of a lossy FR4 PCB material. The measured results of the single array unit show that the maximum radiation direction can be steered from –34 to +34° continuously in the X-cut and –26 to +26° continuously in the Y-cut at 28 GHz. The total dimension of the resulting design package was 18 mm × 14 mm × 0.71 mm. The gain of the array antenna achieved 14.4 dBi and the reflection coefficient of the array antenna was less than −10 dB from 26.5 to 29.5 GHz.

## Figures and Tables

**Table 1:** Maximum scan angle with different antenna spacing.

Maximum Angle *θ_max_* (degree)	Wavelength λ at 28 GHz (mm)	Antenna Spacing *d* (mm)
10	10.71	9.13
20	10.71	7.98
30	10.71	7.14
40	10.71	6.52
50	10.71	6.07
60	10.71	5.74
70	10.71	5.52
80	10.71	5.40

**Table 2:** Comparisons of antenna performance.

References	The Unit Cells Structure	The Bandwidth of Return Loss	The Peak Gain of the Array	Evaluated Peak Gain of the Unit Cell	The Dimensions of the Antenna Module	Material
**[27]**	2 × 2	9.2–10.8 GHz	7.5 dB 10.8–14 GHz	2.5dBi	112 mm × 112 mm	Rogers RT4735LZ
**[28]**	2 × 2	238.4–309.5 GHz	10.1 dB at 71.1 GHz	8 dBi	3 × 1.5 mm²	silicon
**[29]**	4 × 4	57.2–64.5 GHz	6.9 dBi at 62 GHz	7.5 dBi	14 mm × 14 mm × 0.925 mm	Rogers 5880
**[30]**	4 × 4	12 GHz	8.9 dBi at 12 GHz	10.1 dBi	N/A	RO3003
**[31]**	1 × 8	27.2–29.2 GHz	10.33 dBi at 29.2 GHz	6 dBi	130 mm × 42 mm × 0.127 mm	Taconic RF-35
**[32]**	4 × 4	0.8 GHz	3.8 dBi at 30.5 GHz	6 dBi	6.85 × 6.85 cm²	organic
**[33]**	2 × 2	N/A	4.5 dBi at 60 GHz	−1.5 dBi	4.5 mm × 3 mm	RO4003C
**[34]**	1 × 2	9.39–10.26 GHz	N/A	4.8 dBi (Simulated)	15 × 1 5 mm²	RO4003
**[35]**	2 × 32 2 × 2 beamformer chips	23.5–30.5 GHz	EIRP 46 dBm	2~3 dBi	32 elements (5.3 mm) 2 × 2 beamformer (0.5 mm)	Megtron-6
**[36]**	2 × 2	N/A	15 dBi at 20 GHz	9 dBi	2 × 2 Quad-Mode Antenna Array (QMA)	N/A
**[37]**	Yagi–Uda antenna	26.86–28.87 GHz	6.03 dB at 26.86 GHz	6.03 dBi	25 mm × 15 mm	Rogers 5880
**[38]**	2 × 2 × 14	28–30 GHz	EIRP 54dBm	3~4 dBi	70 mm × 70 mm	N/A
**This study**	2 × 2	26.5~29.5 GHz	14.4 dB at 28 GHz	10.6 dBi	18 mm × 14 mm × 0.71 mm	FR4
